# Percutaneous Treatment of Severe Aortic Regurgitation After Surgical Mitral Valve Repair

**DOI:** 10.1016/j.jaccas.2023.102131

**Published:** 2023-11-22

**Authors:** Evelina Toscano, Mahmoud Saad Ahmed, Kush P. Patel, Thomas Treibel, Simon Kennon, Andreas Baumbach

**Affiliations:** a Interventional Cardiology Unit, Policlinico di Monza, Monza, Italy; b Barts Heart Centre, London, United Kingdom; c Institute of Cardiovascular Science, University College London, London, United Kingdom; d Center for Cardiovascular Medicine and Devices, William Harvey Research Institute, Queen Mary University of London, London, United Kingdom

**Keywords:** aortic valve, cardiovascular disease, imaging, mitral valve, treatment, valve replacement

## Abstract

A 54-year-old woman who had recently undergone surgical mitral and tricuspid valve repair was diagnosed with severe aortic regurgitation. She was scheduled for percutaneous treatment and underwent successful transcatheter aortic valve implantation with a 27-mm Trilogy valve (JenaValve Technology). The case documents feasibility of percutaneous treatment in the presence of a mitral ring.

## Central Illustration

**Figure:**
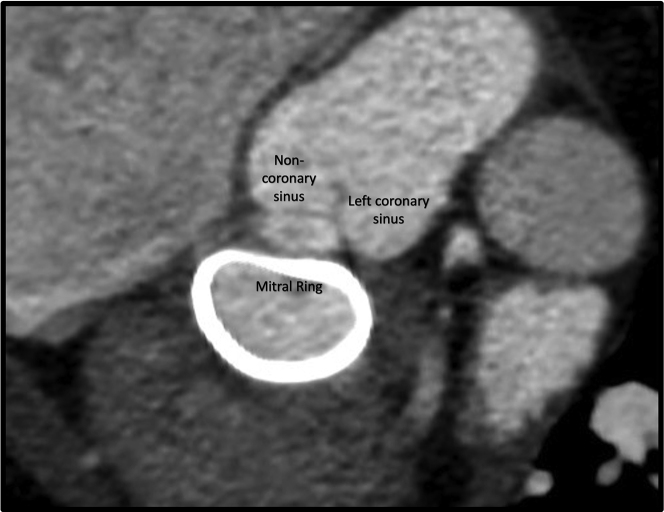

## History of Presentation

This case report describes a 54-year-old woman who was admitted in our institution for worsening dyspnea on NYHA functional class II. Her blood pressure was 130/60 mm Hg, and heart rate was 85 beats/min; her physical examination revealed a holodiastolic murmur and mild basal lung crackles. Her body mass index was 38 kg/m^2^. The patient’s medication consisted of direct oral anticoagulation and furosemide.

**Learning Objectives**

• To learn the etiology of postsurgical AR.
• To understand the treatment options for percutaneous treatment of AR.

The initial laboratory finding revealed a normal white blood cell count, mild anemia, normal renal and liver function, and negative C-reactive protein. Her electrocardiogram showed normal sinus rhythm, normal atrioventricular conduction, and narrow QRS.

## Past Medical History

The patient underwent cardiac surgery for mitral regurgitation (MR) and tricuspid regurgitation in another institution in 2021. The index surgery consisted of mitral valve repair with a 30-mm complete semi-rigid ring implantation, associated with tricuspid repair with ring implantation. The postoperative course was reported uneventful except for paroxysmal atrial fibrillation.

## Differential Diagnosis

Echocardiography on admission revealed trivial MR with normal transvalvular gradient (mean gradient 4.75 mm Hg), a severely enlarged left atrium, normal left ventricle end-diastolic volume with preserved left ventricular ejection fraction, severe aortic regurgitation (AR), moderate tricuspid regurgitation with preserved right ventricular function, elevated pulmonary pressure, and no pericardial effusion (Video 1).

Because there were no previous reports of aortic disease, the differential diagnosis for postsurgical severe AR was between endocarditis and an effect of the surgical intervention.

The patient did not report any clinically overt sign of infection during the index hospitalization or the past months; moreover, the morphology of the valve appeared normal. However, to rule out the diagnosis of infective endocarditis, we performed blood cultures, the results of which were negative for any bacterial growth.^1^

**Figure 1:**
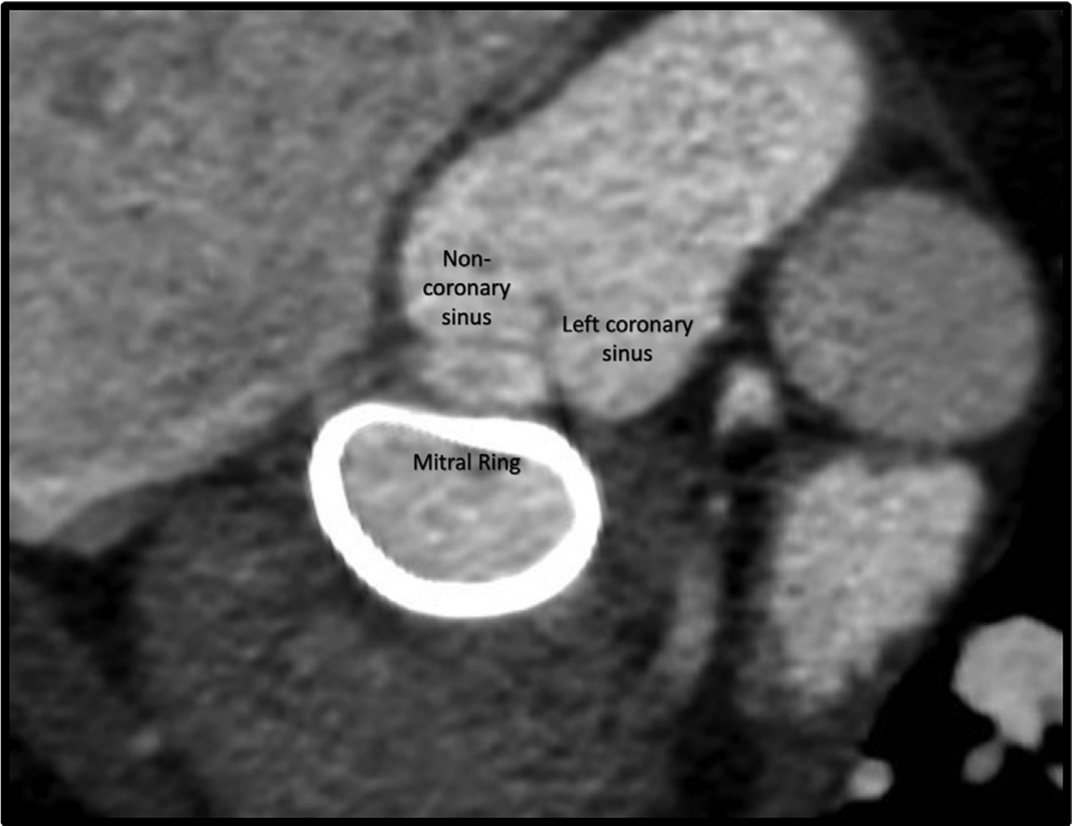
The Aorto-Mitral Curtain Anatomical relation between the left and noncoronary aortic sinuses and the mitral prosthetic ring.

The working hypothesis was that the AR was the consequence of peri-procedural damage of the commissure between the left and noncoronary cusp at the time of the mitral ring suture,^2^ probably a mild aortic commissural pinching with the suture needle when securing the ring to the mitral annulus, causing a trivial AR at the moment of the postsurgical echocardiography. It is possible that the ring had then progressively stretched the pinching, thus causing severe AR in a relatively short period of time (Figure 1).

## Investigations

Transesophageal echocardiography confirmed severe AR in a tricuspid aortic valve with the origin of the regurgitant jet originating from the commissure between the noncoronary and the left coronary cusp. The valve leaflets appeared normal, but the noncoronary cusp appeared mildly pinched (Video 2).

## Management

The case was brought to the Multidisciplinary Heart Team discussion. The surgical option of re-intervention to perform aortic valve replacement was considered technically feasible but at high risk, due to the patient’s previous intervention and her morbid obesity.^3^ A previously reported allergy to warfarin was seen as a further contraindication for a mechanical valve.

**Figure 2:**
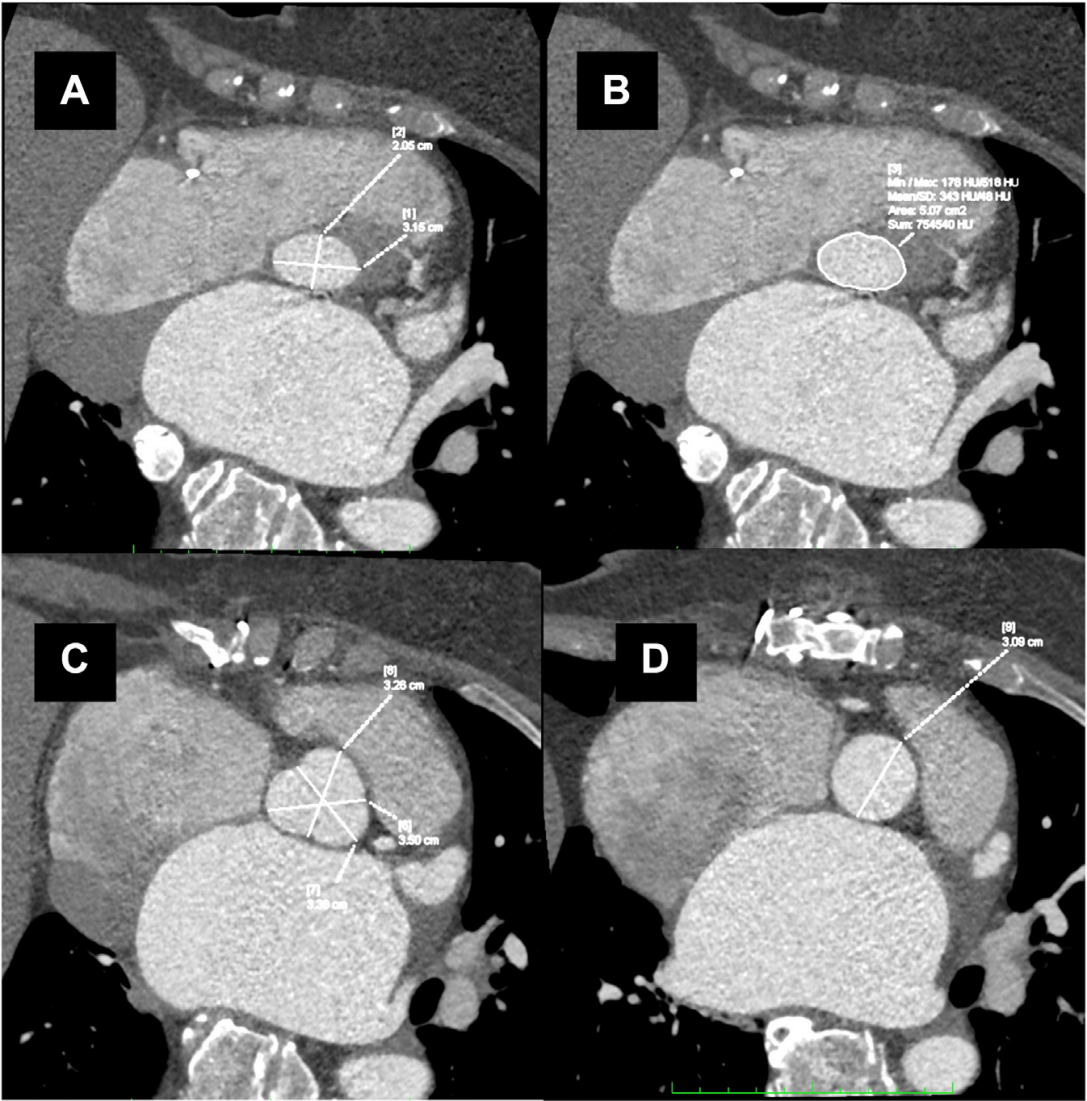
Preprocedural Planning (A) Diameters of the virtual basal ring. (B) Perimeter and area of the virtual basal ring. (C) Sinuses of Valsalva. (D) Sinotubular junction.

The patient underwent a computed tomography (CT) scan to assess the feasibility of a transcatheter procedure, which revealed the following: virtual basal ring (VBR) perimeter of 82.2 mm, VBR diameter of 30 × 19 mm, Valsalva sinuses of 32 × 35 × 33 mm, right and left coronary height >10 mm, and good bilateral femoral accesses (Figure 2). The aortic calcium score was zero.

**Figure 3:**
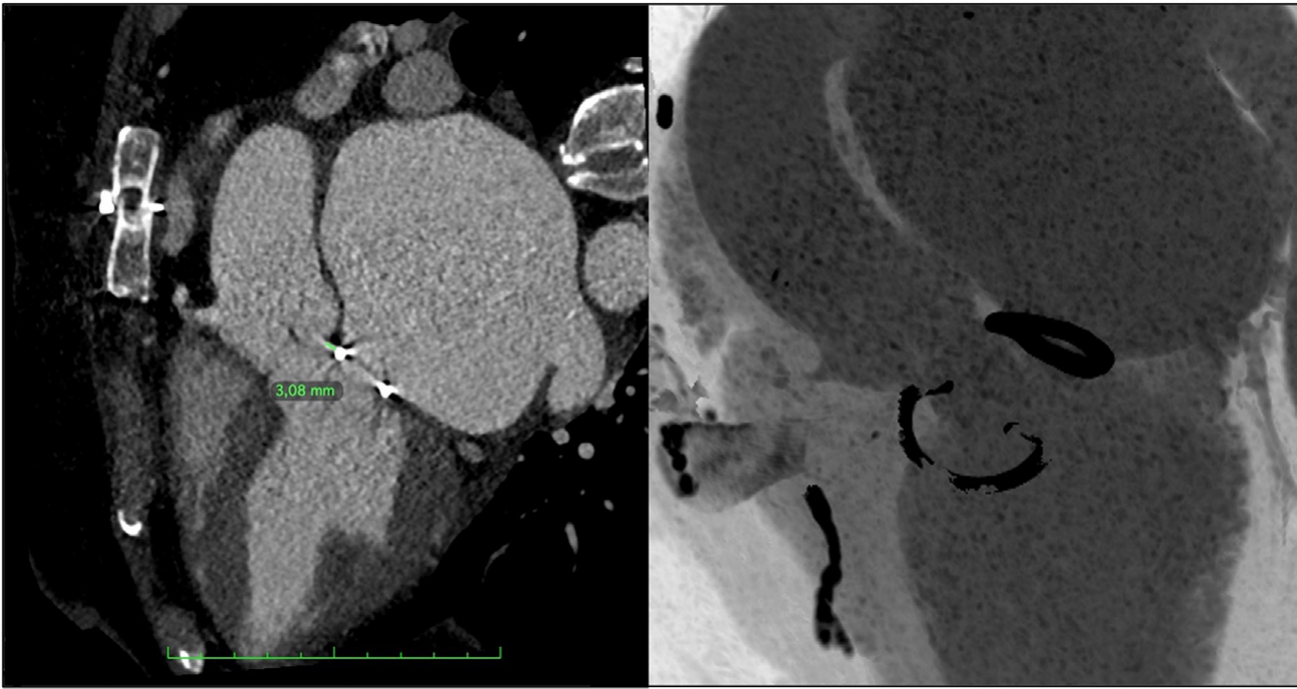
Distance Between the Aortic Annulus and Mitral Ring

The CT analysis raised 2 main concerns: the first was the absolute absence of aortic calcium, which made the option of an off-label implantation of most of the available transcatheter heart valves (THV) unfeasible.^4^ The second concern was the possibility of valve interaction with the mitral ring^5^ as the measured distance from the VBR plane to the ring was only 3 mm (Figure 3).

The only THV with CE mark approval for the treatment of AR is the Trilogy system (JenaValve Technology)^6^; this self-expanding supra-annular THV, available in 3 sizes (23-25-27 mm), is made of a nitinol frame and porcine pericardial tissue. The unique design consists of 2 components: the THV itself, with 3 locators that clip onto the native leaflets, and a sealing ring that protrudes into the left ventricular outflow tract (LVOT) ensuring correct sealing.^7^ The amount of metallic frame protruding into the LVOT is the consequence of implantation depth; in case of optimal implantation depth, the minimum protrusion would be expected to be 3 to 5 mm.

In this specific case, we predicted contact of the valve with the ring but no associated problems with the implantation result. The final heart team decision was for a percutaneous approach. The patient underwent transcatheter aortic valve implantation in November 2022. The procedure was performed under conscious sedation and local anesthesia. A 27-mm THV was correctly implanted in the aortic position, with no paravalvular leak (PVL) and no invasive gradient (Video 3). The electrocardiogram showed mild enlargement of QRS duration (from 76 to 119 milliseconds) but without any further conduction disturbance or AV block.

## Discussion

Severe AR after mitral valve surgery is infrequent, but patients usually experience severe symptoms due to the lack of adaptive remodeling of the left ventricle to the acute valve regurgitation. Differential diagnosis usually includes infective endocarditis and surgical consequence. The multi-modality approach (laboratory tests, transthoracic echocardiography, transesophageal echocardiography, and CT scan) allows clarification of the mechanisms and treatment options for this condition. When treating patients who have undergone previous cardiac surgery, the possibility of a percutaneous approach instead of surgical re-intervention is preferable in most cases; however, there are limited options for transcatheter treatment of pure AR.

The initial international experience with the latest generation Trilogy system allowed the achievement of CE mark approval. The device is becoming a new tool in the portfolio of percutaneous treatment for aortic valve disease.

Initial data from the ALIGNE-AR (A Study to Assess Safety and Effectiveness of the JenaValve Trilogy™ Heart Valve System in the Treatment of High Surgical Risk Patients With Symptomatic, Severe Aortic Regurgitation [AR]) study reported 100% technical success (which consisted of a mean gradient <20 mm Hg and reduction of more than one AR grade), zero conversion to surgery or procedural mortality, 23% rate of permanent pacemaker, and very high rates of none or trace PVL on discharge. Clinical experience with this device, however, is extremely limited, and long-term follow-up data are lacking.^8^

Transcatheter aortic valve implantation in patients with prior mitral valve interventions (ring or prosthesis) usually carries an additional specific risk of device interaction with the mitral structure, which sometimes is only partially predictable.^5^^,^^9^^,^^10^ In particular, an increased risk of THV embolization and increased trans-mitral gradients have been reported, especially in patients with mechanical mitral prosthesis and a distance between the aortic annulus and the mitral prosthesis <7 mm.^5^ In this specific patient, the estimated risk for valve embolization was very low because of the unique leaflet-anchoring mechanism of the selected THV; the major concern was regarding the possible interaction of the mitral ring with the metallic LVOT frame of the THV that could potentially be responsible for frame deformation and paravalvular regurgitation. Proper implantation depth and probably the nonrigid nature of the mitral ring allowed successful implantation with an optimal angiographic and echocardiographic result. Based on this outcome, it is possible to hypothesize that the interaction between this specific THV and a semi-rigid mitral ring is much safer compared with the interaction with a “less flexible” structure, which could be a rigid ring or a mechanical prosthesis. Nonetheless, more evidence arising from expanded use of this THV in different anatomies and clinical scenarios will provide additional parameters to better predict procedural outcomes in patients requiring aortic interventions with prior mitral prosthesis or rings.

## Follow-up

The patient’s postprocedural course was uneventful, and she was discharged on day 4. Predischarge echocardiography revealed correct position and function of the aortic prosthesis (mean gradient <5 mm Hg) with mild PVL, no interaction with the mitral ring, trivial MR with unchanged transvalvular gradient, and preserved left ventricular ejection fraction. At 3 months’ follow-up, the patient was asymptomatic, she experienced marked improvement in exercise tolerance, and echocardiography remained stable.

## Conclusions

Percutaneous treatment of severe symptomatic AR in patients at high surgical risk can be very challenging, as most of the currently available THV are not designed for the treatment of AR, and their use is often off-label. Moreover, when moving outside the clinical trial scenario, it is very common to deal with a higher level of complexity and real-world patient (eg, those with prior valve interventions). Proper patient consent and procedural planning are mandatory to ensure the best clinical outcomes.

The current availability of a new THV specifically tested for patients with AR is going to increase the number of patients at high surgical risk who can receive a percutaneous treatment.

## Funding Support and Author Disclosures

The authors have reported that they have no relationships relevant to the contents of this paper to disclose.

## Appendix

Video 1 Severe Aortic Regurgitation Transthoracic echocardiography on admission revealing a new severe aortic regurgitation and absence of mitral regurgitation.

Video 2 Differential Diagnosis for Severe Aortic Regurgitation Transesophageal echocardiography revealing severe aortic regurgitation originating between the noncoronary and left-coronary sinus of Valsalva, in absence of leaflet damage or signs of infective endocarditis.

Video 3 TAVI Procedure Fluoroscopic view of the 27-mm transcatheter heart valve correctly implanted in the aortic position without any significant paravalvular regurgitation. TAVI = transcatheter aortic valve implantation.
